# Health Behaviors in the Context of Optimism and Self-Efficacy—The Role of Gender Differences: A Cross-Sectional Study in Polish Health Sciences Students

**DOI:** 10.3390/bs15050626

**Published:** 2025-05-03

**Authors:** Małgorzata Dębska-Janus, Michał Rozpara, Agnieszka Muchacka-Cymerman, Paweł Dębski, Rajmund Tomik

**Affiliations:** 1 Institute of Sport Sciences, Academy of Physical Education in Katowice, 40-065 Katowice, Poland; m.rozpara@awf.katowice.pl; 2 Institute of Psychology, Humanitas University in Sosnowiec, 41-200 Sosnowiec, Poland; 3 Department of Psychiatry, Faculty of Medical Sciences in Zabrze, Medical University of Silesia in Katowice, 40-055 Katowice, Poland; p.debski@sum.edu.pl; 4 Department of Tourism and Leisure, Academy of Physical Education in Katowice, 40-065 Katowice, Poland; r.tomik@awf.katowice.pl

**Keywords:** health behaviors, dispositional optimism, self-efficacy, students

## Abstract

This study examines the roles of optimism and self-efficacy in influencing health behaviors among Polish health sciences students, with a focus on gender differences. A cross-sectional design was used with a sample of 318 students. The General Self-Efficacy Scale (GSES), the Life Orientation Test-Revised (LOT-R), and the Health Behavior Inventory (HBI) were used to assess self-efficacy, optimism, and health behaviors’ (HB) intensity, respectively. The variation in the HB was significant concerning the level of dispositional optimism (*F*(2, 312) = 4.22, *p* = 0.016, η^2^p = 0.03). LOT-R results turned out to be a statistically significant condition for higher frequency of positive mental attitude (PMA) behaviors and preventive actions (PAs). Gender differences were observed: higher scores of PMA and PhA were more frequent among men, whilst women were characterized with higher PA. The effect of gender and both GSES and LOT-R for the HBI results was statistically significant in two subscales (with LOT-R for PhA and with GSES for PA). These findings enhance the understanding of psychological determinants of health behaviors and suggest practical implications for educational and public health strategies. Gender turned out to be a significant determinant of some relationships between health behaviors and the examined dispositions in Polish health sciences students.

## 1. Introduction

Although health is one of the most frequently declared supreme values in human life, healthcare varies among people (Ladouceur, 2019; Ryan et al., 2008). Longitudinal studies conducted in the 1970s by M. Lalonde’s team showed that the dominant factor determining the state of human health is lifestyle (Lalonde, 1974). Numerous contemporary studies confirmed this, attributing different elements of lifestyle (health behaviors) as key means of primary prevention, modulators of genetic potential, and environmental influences (Francesconi et al., 2023; Friedman, 2020; Sadiq, 2023; Santos, 2022; World Health Organization, 2004). According to the results of international reports and research, the percentages of people meeting health recommendations for key health behaviors, like regular physical activity, reasonable diet, or sleep hygiene, are low (European Commission, 2018, 2022; Ministerstwo Sportu i Turystyki, 2021). The variability in adherence to health recommendations remains a significant public health challenge. International self-reported studies indicate that only 5.8% of European adults lead a healthy lifestyle (Marques et al., 2019), 69% of populations declare to meet the minimal recommended dose of physical activity (World Health Organization & Organization for Economic Co-Operation and Development, 2023), and 65% of Americans report getting enough sleep (Ramar et al., 2021). Self-efficacy and optimism have been identified as critical psychological determinants of health behaviors, influencing both the initiation and maintenance of such behaviors (Buchwald & Hobfoll, 2021; Schwarzer & Łuszczyńska, 2022; Warner & Schwarzer, 2025).

The difficulty in maintaining health-promoting behaviors is linked to the necessity of keeping them regular and the ability to forgo immediate pleasures in favor of delayed gratification (Bishop, 1994). A healthy lifestyle requires discipline and good time management (Rovniak et al., 2002). In seeking the reasons behind individual differences in health-promoting activities, the cognitive-behavioral approach dominates. To understand the varying levels of healthcare within society, it is essential to consider health behaviors together with their associated cognitive elements (Bishop, 1994). The models developed so far to explain health behaviors are primarily based on social learning and attribution theory. Some theoretical models concerning health behaviors focus on the process of forming the intention to engage or modify activity and its determining factors, such as motivational models—Health Belief Model by Rosenstock et al. (1959) and the Theory of Reasoned Action (Ajzen, 1991), while others concentrate mainly on explaining the process of realizing the intentions and mechanisms of their continuation (post-intentional models: Self-Determination Theory (Ryan & Deci, 2000), Transtheoretical Model of Health Behavior (Prochaska & DiClemente, 1982), and the Health Action Process Approach (Schwarzer, 1992)). As people often do not behave in accordance with their intentions, a need to explain the intention–behavior gap appeared (Sheeran & Webb, 2016).

This study aims to explore the relationship between optimism, self-efficacy, and health behaviors among health sciences students, with a particular focus on gender differences. Self-efficacy reflects an individual’s confidence in their ability to perform specific tasks. Optimism, defined as the tendency to expect favorable outcomes, serves as a psychological buffer, enhancing persistence in the face of challenges (Carver et al., 2010). Both are relatively stable dispositions but can be shaped and changed. Gender differences in these constructs have been documented, with men often exhibiting higher levels of self-efficacy and optimism in certain contexts (Lange et al., 2018).

This study focuses on the analysis of the relationships between undertaking health behaviors and two social-cognitive dispositions in the context of the categories of health behaviors. It is worth noting that so far, there are no Polish research reports that simultaneously verify these two resources in the context of undertaking general health behaviors. It is important to emphasize that, even in European studies, researchers tend to examine the relationship between health behaviors and only a single resource, and rarely assess gender as a moderating factor (Kupcewicz et al., 2022b; Scheier et al., 2021; Trudel-Fitzgerald et al., 2019). There are relatively few international studies that simultaneously examine these resources in the context of health behaviors; however, their findings indicate significant associations between optimism, self-efficacy, and health behaviors among students from Pakistan (Mohsin et al., 2023) and Iran (Beyrami et al., 2015). Furthermore, the studies published to date have not focused on examining the role of optimism and general self-efficacy within certain groups of health-enhancing behaviors. In our work, we verified the role of these two dispositions in the frequency of undertaking behaviors related to both physical and mental health. We also considered these relationships from the perspective of four groups of behaviors: those related to mental healthcare, physical health practices, proper eating habits, and preventive behaviors. Based on the current state of knowledge on the relationship between optimism, self-efficacy, and healthcare presented in the Introduction, we expected that the level of these dispositions would significantly differentiate the intensity of health-related behaviors, and that this differentiation may depend on the types of these behaviors and the gender of the respondents. The findings may contribute to the understanding of psychological factors in health behaviors and suggest implications for educational and public health interventions.

## 2. Literature Review

This study is grounded in the processual model, which represents a contemporary and integrative approach to understanding the mechanisms underlying health behaviors. The Health Action Process Approach (HAPA) developed by R. Schwarzer is based on Bandura’s Social Cognitive Theory and considers the health behavior formation (Schwarzer, 2016). According to Bandura (1977), simply believing that an action will lead to the desired goal (outcome expectation) is not enough to undertake it. The intention–behavior gap in health behaviors needs more proximal factors that might compromise or facilitate its translation. The key factor in HAPA is the belief that one is capable to perform a specific action and persist in achieving the intended goal in a particular situation, expressed by self-efficacy (Lange et al., 2018). It is a relatively stable disposition that can be characterized by level, degree of generality, and strength. The level is related to the difficulty of the behavior—the belief in efficacy can concern only easy actions or quite complicated ones, or both types. Generality is associated with the area of actions—efficacy can concern diverse actions or a specific category (e.g., learning). Strength is a measure of persistence in action. Self-efficacy is an important determinant of health behavior both in its motivation and volition phase. According to Schwarzer, phase-specific self-efficacy plays a major role in the volition phase of health behaviors (Schwarzer, 2016). The intention to undertake or change a particular health behavior does not give a guarantee of its performance. In order to implement pro-health change, intention has to be transformed into detailed instructions of how to perform the desired action. The volition process is strongly influenced by general self-efficacy, as the number and quality of action plans and their control are dependent on one’s perceived competence and experience. The Health Action Process Approach (HAPA) emphasizes the distinction between motivational and volitional phases of health behavior. While the motivational phase involves intention formation, the volitional phase concerns action and maintenance. This study focuses on the latter, examining how self-efficacy and optimism (outcome expectation) translate into observable health behaviors. When health behavior is being performed, self-efficacy determines the amount of effort invested and the perseverance of pro-health change (Schwarzer & Łuszczyńska, 2022) Research indicates a positive relationship between the intensity of generalized self-efficacy and the pursuit of ambitious goals and commitment to achieving them (Bandura & Wood, 1989; Fort et al., 2011; E. A. Locke & Latham, 1990). Recent studies, such as those by Lizarte Simón et al. (2024a, 2024b), highlight the significant role of self-efficacy in academic engagement, coping with challenge/obstacle stressors, and technology-related behaviors, such as cyberloafing and smartphone addiction. These findings provide a broader perspective on self-efficacy as a universal determinant of behavior across various domains. This relationship is also observed in health-related behaviors (Cholewa et al., 2015; Doumit et al., 2022; Gacek, 2016; Hamerman et al., 2023; Kusol & Kaewpawong, 2023; Schwarzer et al., 2011; Schwarzer & Fuchs, 1995; Sekuła et al., 2019). They mainly concern the role of self-efficacy as a mediator of primary preventive actions (e.g., using seat belts and breast self-examination) (Bill et al., 2023; Luszczynska, 2004; Syrjälä et al., 2001), maintenance of recommended pro-health activities in patients (physical activity, diet, and sleep hygiene) (S. R. Locke et al., 2020; Luszczynska et al., 2007; Schwarzer, 2008; Schwarzer et al., 2011; Sekuła et al., 2019), and using pro-adaptive coping strategies in difficult situations (COVID-19 pandemic) (Doumit et al., 2022; Hamerman et al., 2023; Kusol & Kaewpawong, 2023). Previous publications conducted in Poland most frequently examine the relationship between healthcare and self-efficacy in sick people and seniors.

Maintaining health is based on the ability to adapt to environmental, psychological, and social stimuli in a way that does not disrupt the structure of the organism and functioning in the biopsychosocial dimension (Aleksandrowicz, 1972). According to the Conservation of Resources Theory by Hobfoll, the effectiveness of this adaptation process depends, among other things, on the individual’s resources that influence motivation to protect and develop health, limit activities generating health risks, and, in case of health loss, support the recovery process (Hobfoll, 2002; Hobfoll et al., 2018). Among the psychological properties conducive to health, alongside the described self-efficacy, optimism is also mentioned. Most generally, it can be defined as a disposition associated with a positive attitude toward life. According to a comprehensive definition, optimism is a relatively stable tendency to perceive, explain, and evaluate the world and phenomena occurring in it in positive rather than negative terms, and a relatively stable inclination to anticipate and expect future, more or less specific events as favorable rather than unfavorable (Stach, 2006). Carver and Scheier introduced the concept of dispositional optimism as a regulatory mechanism determining the choice of goals and directing activity toward their realization. According to the authors, this disposition expresses the expectation of positive future events and the belief that unfavorable events will occur sporadically or not at all (Carver & Scheier, 2014; Scheier & Carver, 2018). Referring to health behaviors, optimism can be significant both in the motivational phase when forming intentions related to health-promoting activities and in the volitional phase—continuing these activities, especially when obstacles arise. It should be mentioned that in the intention formation phase, an important cognitive factor is outcome expectation, in which, generally, positive expectations strongly influence positively. Majority of previous studies confirm the positive relationship between optimism and health (Adorni et al., 2021; Dionigi et al., 2020; Fasano et al., 2020; Rasmussen et al., 2009; Scheier et al., 2021). On the contrary, some researchers indicated that preventive behaviors are associated with lower optimism (Adorni et al., 2021). International pools indicated the relationship between optimism and healthcare, too (Celano et al., 2020; Giltay et al., 2007; Kronrod et al., 2022; Kupcewicz et al., 2022b; Steptoe et al., 2006; Trudel-Fitzgerald et al., 2019). In Poland, such studies mainly concern the relations between optimism and effectiveness of treatment in selected diseases and healthy aging (Gacek, 2016; Ślusarska, 2012; Ślusarska et al., 2012; Zadworna & Ogińska-Bulik, 2013). The study of optimism among students in the context of health behaviors was undertaken by Kupcewicz et al., who confirmed a positive relationship between them in nursing students (Kupcewicz et al., 2022a).

Gender differences in health behaviors (Adorni et al., 2021; Mohsin et al., 2023; Ślusarska et al., 2012; Sood et al., 2019; Thompson et al., 2016) and their social-cognitive predictors (Celano et al., 2020; Ślusarska, 2012; Zadworna & Ogińska-Bulik, 2013) are being emphasized in domestic and foreign publications more frequently. According to the results of most research, women are more likely to follow a healthy lifestyle and to be characterized with greater optimism levels, whilst men tend to have higher levels of self-efficacy. This is when health behaviors are assessed in general, but when health habits are assessed in detail—by area (e.g., physical activity, sleep hygiene, nutrition, and stress management)—these trends tend to differentiate more (Buchwald & Hobfoll, 2021; Scheier et al., 2021; Schwarzer & Fuchs, 1995). There is still little research in this area to systematize the directions of the above-mentioned differences. At the same time, Lange et al. (2018) emphasized the importance of gender differences in undertaking health behaviors for effective promotion of a healthy lifestyle.

Based on the above understanding, we propose the following research hypotheses:

**H_1_:**  *The level of self-efficacy and dispositional optimism is a factor differentiating the frequency of health behaviors.*

**H_2_:**  *The relations between health behaviors and examined social-cognitive dispositions will vary according to the category of behaviors (proper eating habits, preventive actions, pro-health activities, and positive mental attitude).*

**H_3_:**  *Gender is a moderating factor in the relationship between self-efficacy and health behaviors, as well as between dispositional optimism and health behaviors.*

## 3. Materials and Methods

### 3.1. Study Design and Participants

A cross-sectional study was conducted on Polish health sciences students. It included students enrolled in health-related programs from the area of southern Poland in the year 2023. The following inclusion criteria were applied: consent to participate in the study (a), completion of the full research program (b), being a student of a health sciences program (c), and no diagnosed mental disorders within the past year (d). The study group consisted of 318 students aged 18–24 years (19.6 ± 1.1 years), with 188 women (59.1%) and 130 men (40.9%).

### 3.2. Setting

The research was conducted in direct contact, in an auditory format, using electronic tools during meetings with the respondents. Each respondent participated in three such meetings. In total, 30 diagnostic sessions were conducted as part of the study.

The research procedure was approved by ethical approval from the Research Ethics Committee of the Medical University of Silesia in Katowice (PCN/0022/KB/277/19). Before data collection, all participants were informed about the aim of the study. They voluntarily participated in the research and had the right to withdraw their participation at any time. Written consent was obtained from each participant for the use of the collected examination.

### 3.3. Variables and Instruments

Psychological resources (independent variables—IVs) were verified using the General Self-Efficacy Scale (GSES) and the Life Orientation Test-Revised (LOT-R), while the intensity of health behaviors (dependent variable—DV) was diagnosed using the Health Behavior Inventory Scale (HBI). During the interviews, data were also obtained on respondents’ gender (women and men), as an effect modifier.

The General Self-Efficacy Scale (GSES) was developed based on Bandura’s concept of expectations and self-efficacy (Bandura, 1977). The Polish adaptation of the tool was performed by Juczyński (2012). The tool can be used to diagnose a person’s general belief in their ability to cope with difficult and problematic situations and to carry out various activities. The scale measures a uniform construct—the Self-Efficacy Index. According to the factor analysis, no subscales were identified. The GSES consists of 10 statements, which the respondent evaluates on a 4-point scale, where 1 means no, 2—rather no, 3—rather yes, and 4—yes. The overall self-efficacy index is the sum of the points for the responses given according to the questionnaire key. The raw score ranges from 10 to 40 points and is standardized to a Sten scale according to the instructions. The reliability of the GSES, determined by Cronbach’s alpha coefficient, is 0.85 (Juczyński, 2012). For the studied group, the reliability coefficient is 0.85, 95% CI [0.83, 0.88].

The Life Orientation Test-Revised (LOT-R) questionnaire is used to verify the level of dispositional optimism according to the theory of Scheier and Carver (1985). The Polish adaptation of this tool was developed by Poprawa and Juczyński (Juczyński, 2012; Poprawa, 1996). This tool measures the general level of dispositional optimism, and the factor analysis did not reveal the existence of subscales. The questionnaire consists of 10 statements, which the respondent evaluates on a scale from 0 to 4, where 0 means does not apply to me, and 4 means applies to me. Six items are diagnostic, and the remaining four are buffer items. The overall level of dispositional optimism is calculated by summing the points assigned to specific responses from the six diagnostic items according to the key. The score ranges from 0 to 24 points. Norms for the scale are developed on a Sten scale for adults without gender division and without distinguishing age groups. The reliability of the tool, determined by Cronbach’s alpha coefficient, is 0.76 (Juczyński, 2012), while for the studied group, the coefficient is 0.65, 95% CI [0.59–0.71].

The Health Behavior Inventory (HBI) by Juczyński (2012) is based on Gochman’s theory (Baumgart et al., 2015) and is intended for studying adults, both healthy and sick. The tool allows to verify the General Health Behaviors Indicator (HB) and its four subscales: positive mental attitude (PMA; avoiding strong emotions, tension, and stressful and depressing situations), proper eating habits (PEH; type and frequency of food consumed), preventive actions (PAs; following health recommendations and obtaining information about health and illness), and pro-health activities (PhA; everyday habits: sleep, recreation, and physical activity). The HBI consists of 24 statements regarding the frequency of healthy behaviors. The intensity of individual behaviors is assessed on a 5-point Likert scale, where 1 means almost never, 2—rarely, 3—occasionally, 4—often, and 5—almost always. The responses are then converted into points and summed according to the key. Cronbach’s alpha coefficient for the entire scale is 0.80, while for individual subscales it ranges from 0.60 to 0.65 (Juczyński, 2012). The reliability of the tool in the studied group is 0.77, 95% CI [0.73, 0.81]. Sten norms for adults have been developed separately for men and women only for the main scale.

### 3.4. Study Size

The general population consisted of 1025 students. The calculation of the minimum sample size using the Daniel and Cross formula (Daniel & Cross, 2018), assuming a 5% margin of error and a 50% structure ratio, amounted to 280 individuals. Considering the possibility of a reduction in the final number of participants due to the inclusion criteria, a 25% surplus was adopted. The study began with 350 students, but 5.7% were excluded due to not completing the full program (criterion b), and another 3.4% due to the declaration of mental disorders (criterion d). The study group consisted of 318 students aged 18–24 years (19.6 ± 1.1 years), with 188 women (59.1%) and 130 men (40.9%).

### 3.5. Statistical Methods

Basic descriptive statistics parameters were determined as means (*M*), standard deviations (*SD*), frequencies (*f*), and relative frequencies in percentages (*rf*). The normality of variable distributions was verified using the Shapiro–Wilk test, *W*, and the homogeneity of variances using the Levene test, *F*. The reliability of the measures used was assessed by calculating Cronbach’s α coefficients with a 95% confidence interval in the study sample. The variation in the intensity of health behaviors (DV) among students with different levels of self-efficacy, dispositional optimism (IVs), and gender as an effect modifier was verified based on a two-way analysis of variance (ANOVA) and Bonferroni post hoc tests. Effect size by partial eta squared (η^2^p) was also calculated. The calculations were performed using IBM SPSS Statistics (Version 26.0).

## 4. Results

The average GHB was 79.4 ± 10.8 pts. Nearly half of the surveyed students had an average intensity of health behaviors (47%), 28% had a high intensity, and the remaining 25% had a low intensity. The respondents showed the greatest care for health in PMA (20.6 ± 3.7 pts.) and the least in the PEH subscale (19.1 ± 4.1 pts.).

The average level of self-efficacy was 27.5 ± 5.0 pts. It was not significantly different between genders (*t*(316) = 1.83, *p* = 0.068, *d* = 0.21). The GSES scores for men (28.1 ± 5.0 pts.) were higher compared to women (27.1 ± 5.0 pts.). Among the respondents, the majority had an average sense of self-efficacy (44%), 32% had a high level, and the remaining 24% had a low level of this disposition.

The average optimism score was 14.5 ± 3.9 pts., with a higher value observed in men (15.2 ± 3.6 pts.) versus women (14.1 ± 4.0 pts.). The Student’s *t*-test showed significant differences in LOT-R results between women and men (*t*(316) = 2.35, *p* = 0.019, *d* = 0.27). The largest percentage of respondents (39%) had an average level of functional optimism. Pessimistic students constituted 31%, and optimistic students 30% of the total respondents.

**Figure 1:**
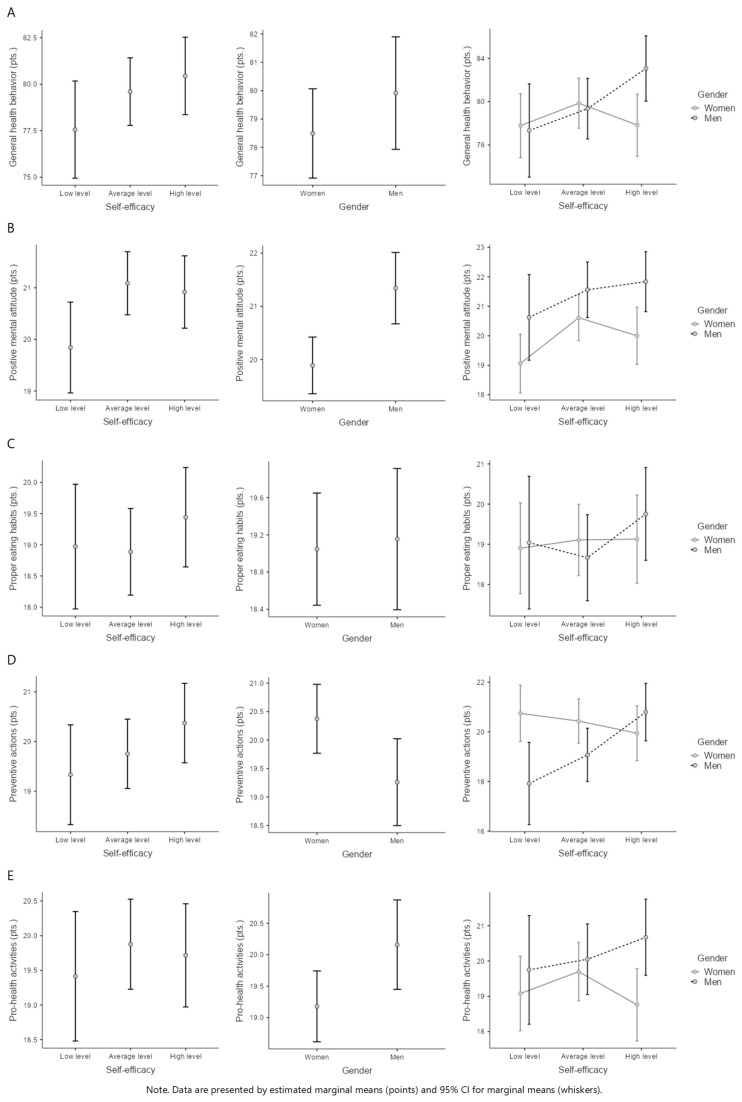
Health behaviors across different levels of self-efficacy and gender. (**A**)—General health behaviors vs. self-efficacy and gender, (**B**)—Positive mental attitude vs. self-efficacy and gender, (**C**)—Proper eating habits vs. self-efficacy and gender, (**D**)—Preventive actions vs. self-efficacy and gender, (**E**)—Pro-health activities vs. self-efficacy and gender.

A two-way analysis of variance did not show a significant difference in the intensity of general health behaviors between groups of respondents with low, average, and high levels of self-efficacy (*F*(2, 312) = 1.47, *p* = 0.231, η^2^p = 0.01), and between women and men (*F*(1, 312) = 1.22, *p* = 0.270, η^2^p < 0.01). There were no statistically significant differences in GHB for the interaction effect between gender and self-efficacy factors (*F*(2, 312) = 2.41, *p* = 0.091, η^2^p = 0.02). However, there was a tendency for a higher score among men with higher GHB and students with moderate and high levels of self-efficacy compared to those with low levels (Table 1 and Figure 1A).

Gender comparisons of the HBI subscale scores showed significant differences in the categories of positive mental attitude (*F*(1, 312) = 11.15, *p* < 0.001, η^2^p = 0.03), preventive actions (*F*(1, 312) = 5.06, *p* = 0.025, η^2^p = 0.02), and pro-health activities (*F*(1, 312) = 4.52, *p* = 0.034, η^2^p = 0.01). Higher scores of PMA and PhA were more frequent among men, whilst women were characterized with higher PA (Table 1 and Figure 1B,D,E).

The interaction effect of gender and self-efficacy for the PA variable was statistically significant (*F*(2, 312) = 4.32, *p* = 0.014, η^2^p = 0.03), but the Bonferroni post hoc test did not confirm the results of the *F* test. The differences in preventive actions in men and women with a high level of self-efficacy were not statistically significant (*p* > 0.05; Table 1 and Figure 1D). However, a clear trend was observed, indicating a divergent direction of the interaction between preventive actions (PA) and general health behaviors (GHBs) across genders. Among men, the intensity of health-promoting behaviors increased with higher self-efficacy, whereas among women, the opposite pattern was noted (Figure 1).

**Figure 2:**
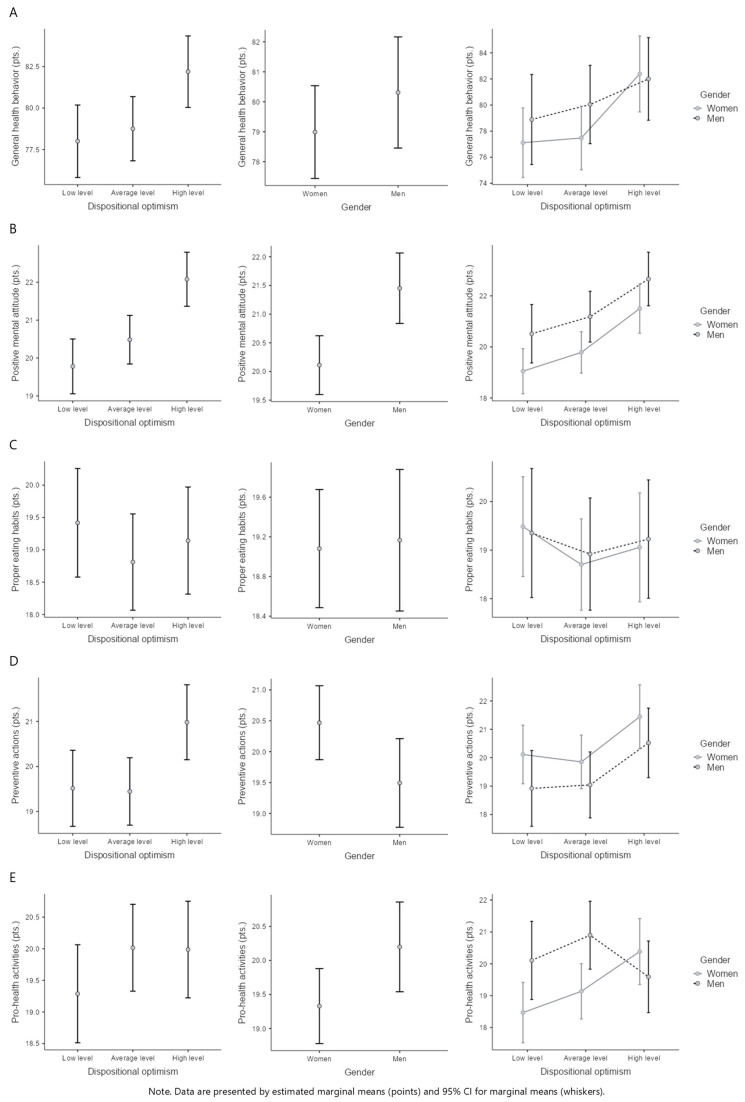
Health behaviors across different levels of dispositional optimism and gender. (**A**)—General health behaviors vs. self-efficacy and gender, (**B**)—Positive mental attitude vs. self-efficacy and gender, (**C**)—Proper eating habits vs. self-efficacy and gender, (**D**)—Preventive actions vs. self-efficacy and gender, (**E**)—Pro-health activities vs. self-efficacy and gender.

A two-way ANOVA showed a statistically significant difference (*F*(2, 312) = 4.22, *p* = 0.016, η^2^p = 0.03) in the intensity of general health behaviors between participants with low, average, and high levels of dispositional optimism. Higher scores of GHB appeared more frequently in students with high levels of dispositional optimism versus those with low levels (*p =* 0.023; Figure 2A). There were no statistically significant differences in GHB for an interaction effect between gender and dispositional optimism factors (*F*(2, 312) = 0.52, *p* = 0.593, η^2^p < 0.01; Table 2 and Figure 2A).

LOT-R results turned out to be a statistically significant condition for positive mental attitude behaviors (*F*(2, 312) = 10.54, *p* < 0.001, η^2^p = 0.06) and preventive actions (*F*(2, 312) = 4.37, *p* = 0.013, η^2^p = 0.03). Subjects with a high level of dispositional optimism had a higher intensity of PMA vs. participants with average (*p* = 0.004) and low levels (*p* < 0.001) of dispositional optimism (Figure 2B). Students with a high level of dispositional optimism had higher intensity of PA vs. those with average (*p* = 0.046) and low levels (*p* = 0.022) of dispositional optimism (Figure 2D).

In the study group, PhA was determined both by gender and regulated by the respondents’ dispositional optimism (*F*(2, 312) = 3.57, *p* = 0.029, η^2^p = 0.02). Pro-health actions in women with low dispositional optimism were lower than in men with an average level of dispositional optimism (*p* = 0.022; Table 2 and Figure 2E).

## 5. Discussion

Despite the well-verified empirical knowledge about the health-promoting impact of basic health behaviors and health policies aimed at their popularization, people’s attention to health is highly variable. In seeking the causes of individual differences in health-promoting activity, the cognitive-behavioral approach predominates. Most models explaining the adoption and change of behaviors emphasize the role of cognitive factors, which are important both in the phase of forming intentions for health-promoting activities and in maintaining them. Additionally, the importance of individual resources supporting continuation of health practices in the face of stress and life difficulties has been emphasized (Hobfoll, 2002). According to The Conservation of Resources Theory by Hobfoll, self-efficacy and optimism are among dispositions that support the maintenance of health behaviors by counteracting the fatigue accompanying the implementation of these behaviors (Hobfoll et al., 2018). Therefore, the aim of this study was to verify the relationship between the intensity of health behaviors and self-efficacy as well as dispositional optimism in a group of young adults—students.

### 5.1. Psychological Resources and Health Behaviors in the Study Group with Respect to Previous Research Findings

The average GSES score in this study (27.5 ± 5.0 pts.) was slightly lower compared to that reported by Nowak et al. (2018) among dietetics students and in the study of medical students conducted by Borek et al. (2016). However, it was very similar to the score obtained by social sciences students from another university. The average LOT-R score was 14.46 points, which is similar to the results obtained in young adults in another study conducted Poland (Chodkowski, 2023; Juczyński, 2012) and lower compared to students from other European countries (Kupcewicz et al., 2022a). With reference to health behaviors, optimism may be important both in the motivational phase, when forming intentions related to health-promoting activities, and in the volitional phase, when continuing these activities, especially in the face of obstacles. The average intensity of health behaviors in this study was 79.98 ± 10.71 points and was not significantly different between genders. This result is similar to that obtained in Polish studies conducted among adults (Juczyński, 2012) and students from different universities (Kropornicka et al., 2015; Kupcewicz et al., 2022a, 2022b; Nowak et al., 2018). In comparison to the students from Spain or Hungary, Polish students’ scores were a bit lower (Kupcewicz et al., 2022a; Lesińska-Sawicka et al., 2021).

A significant gender difference in LOT-R results was observed. Men were characterized with higher dispositional optimism, in line with research conducted in different populations and various areas of optimistic beliefs (Bjuggren & Elert, 2019; Dawson, 2023; Jacobsen et al., 2014; Lin & Raghubir, 2005). The explanation of gender differences in optimism has received relatively little attention in the literature. In our study, it may be connected with the culture and traditional patriarchal division of roles and responsibilities, which has only been changing in our country in the last decade. This outcome may also be attributed to the specific characteristics of the study group, which consisted of health sciences students. Within this group, a positive attitude toward health behaviors can be expected, both in the cognitive and emotional domains, which may, in turn, translate into the behavioral component. In such a homogeneous group, characterized by a high level of awareness regarding the impact of various health behaviors on psychophysical functioning, the degree of engagement in health-promoting behaviors is more likely to be differentiated by internal factors, such as the psychological resources under study, emotional state, as well as social factors, such as social support. In this research, the GSES was not statistically differentiated by gender, but a tendency toward higher scores in men compared to women was observed. Similar trends were also observed in US adults and adolescents (Robinson et al., 2022; Sood et al., 2019), as well as in a group of Albanian students (Shkullaku, 2013).

In most national and international studies on the overall intensity of health-promoting behaviors, women tend to exhibit higher healthcare. In our study, this tendency was reversed—men were characterized by a higher intensity of health behaviors. It must be emphasized that our study group consisted of health sciences students whose health literacy and positive attitude toward a health-enhancing lifestyle seemed to be high regardless of gender, whilst in the general population these factors are considered to be higher in women. However, in the detailed diagnosis of specific behaviors related to physical healthcare, such as physical activity, rational diet, and sleep (health practices and eating habits), men achieve better results (Adorni et al., 2021). Conversely, activities aimed at preventive behaviors are performed more frequently by women, and this tendency is in line with the results of our study (Knapova et al., 2024; Lange et al., 2018).

It turned out that significant differences in the intensity of HB appeared only with respect to optimism. The intensity of health behaviors was significantly higher in groups with moderate and high LOT-R scores compared to students with a low level. This relationship was also confirmed by studies in cardiac patients (Łatka et al., 2013) and women with alcohol dependence disorder (Chodkowski, 2023). The level of optimism was a significant factor primarily in behaviors connected with positive mental attitude and preventive actions. Students with moderate and high levels of optimism participated in these behaviors significantly more frequently than those with low LOT-R results. The trend toward higher HBI in the groups of subjects with a moderate and high LOT-R result was also observed in the case of the pro-health activities subscale. Understanding dispositional optimism as a mechanism for regulating goal selection and directing activity toward their realization, it is particularly important in all actions whose results are not immediate and require delayed gratification, which is typical for health behaviors. A high level of dispositional optimism promotes the formation of intentions for health-promoting activities and their continuation, especially in the face of obstacles, as confirmed by the results of this study. The relationship between the level of optimism and healthcare has also been demonstrated in comparative studies of nursing students from Poland, Slovakia, and Spain (Kupcewicz et al., 2022b).

The GSES level did not differentiate HB at a statistically significant level, but a tendency to take greater care of health in groups with a higher GSES result compared to students with a low level was observed. Also, at the subscale level, there were no significant differences in HB between groups of students with high, moderate, and low self-efficacy. However, there was a tendency for a higher intensity of preventive behaviors in subjects with moderate and high levels of optimism compared to those with low levels of this disposition. Self-efficacy could be a factor protecting against the negative consequences of stress, eliminating the negative impact of overload at work (Fürtjes et al., 2023; Zhang et al., 2024), and also correlates with the regularity of participation in preventive procedures (Zhang et al., 2024).

### 5.2. Dispositional Optimism and Self-Efficacy as Determinants of Health Behaviors

This is the first study to simultaneously verify general self-efficacy and dispositional optimism in the context of an overall healthy lifestyle. Only the relations between these two social-cognitive factors and certain groups of health behaviors, like preventive activities, performing physical activity, or undertaking risky health behaviors, have been checked and partially confirmed before (Hajek & Helmut-König, 2017; Mohsin et al., 2023; Waldrop et al., 2001). The explanation of relations between optimism, self-efficacy, and the intensity of health behaviors may lie in the nature of health behaviors, which require systematic action to achieve results. Despite beliefs in the ability to meet them in the life of a contemporary, busy person, situations may arise where the continuation of behavior must be temporarily halted or its execution method liberalized to adapt to current capabilities, e.g., time, physical condition, or health. In such situations, the dominant protective factor in the context of maintaining behavior is the disposition to expect a positive result of health activity despite temporarily stopping its implementation, like dispositional optimism. Therefore, it may be especially important in the volitional phase of behavior change. However, self-efficacy is the base for formulating intentions of health-promoting activities and, therefore, it is especially important in the motivational phase of the decision to change or undertake behavior and to start the process. The findings of this study align with the broader understanding of self-efficacy as a determinant of various behaviors. For instance, Lizarte Simón et al. demonstrated that self-efficacy significantly influences academic engagement by mitigating anxiety and enhancing psychological well-being (Lizarte Simón et al., 2024a). Similarly, self-efficacy has been shown to moderate the impact of challenge/obstacle stressors in vocational education, as well as behaviors like cyberloafing, with smartphone addiction acting as a mediator (Lizarte Simón et al., 2024b). These insights suggest that the mechanisms of self-efficacy extend beyond health behaviors, impacting performance and adaptation in diverse environments.

### 5.3. Gender as a Modifier of the Relationship Between Self-Efficacy, Dispositional Optimism, and the Intensity of Health Behaviors

Additionally, the results of this study pay attention to gender as a factor modifying the relationship between GHB and the examined social-cognitive dispositions. It was a significant factor only in the case of the preventive actions (GSES) and pro-health activities (LOT-R) subscales. A clear trend was observed, suggesting a divergent direction of the interaction between PA and GHB across genders, although this difference was not statistically significant. Among men, the intensity of preventive actions increased with higher self-efficacy, whereas among women, the opposite pattern was noted, suggesting that self-efficacy may play a gender-specific role in shaping health behaviors. The frequency of undertaking activities in the field of PhA increased significantly in relation to the LOT-R level in women, while in men it increased to a moderate level and then decreased. For the remaining areas of health behaviors, gender was not a significant modifier of relations with the examined dispositions, however, tendencies toward a different course of these relationships were observed. This is probably related to the previously described gender differences in the intensity of health behaviors. In our study, significant gender differences appeared in the case of behaviors related to a positive mental attitude and pro-health activities. They were also confirmed in other studies (Adorni et al., 2021; Crimmins et al., 2011; Knapova et al., 2024; Lange et al., 2018; Zadworna & Ogińska-Bulik, 2013). The influence of gender on health behaviors, while significant in some subscales, showed inconsistencies that may reflect sociocultural nuances or sample-specific characteristics. Although the observed effect sizes (η^2^p ≤ 0.03) were small, they remained meaningful in the context of psychological research, where incremental contributions often have cumulative practical relevance. This is particularly true in exploratory studies like ours, which aim to identify patterns that can guide future, more targeted investigations. It is important to note that the HAPA model, selected as the theoretical framework for this study, has certain limitations. It tends to underestimate the influence of emotional factors, such as fear and anxiety, and does not fully account for cultural diversity, gender-specific differences, or situational barriers impacting health behavior change. Additionally, contextual barriers, such as resource limitations or access to health services, are not sufficiently addressed. To gain a more comprehensive understanding of health behaviors, future studies should consider integrating complementary approaches, such as the Theory of Planned Behavior or Social Cognitive Theory.

These findings suggest the need for a deeper exploration of how gender roles and cultural factors shape health behavior patterns. Future studies could investigate these dynamics through qualitative approaches or stratified analyses. At the same time, Lange et al. (2018) emphasized the importance of gender differences in undertaking health behaviors for effective promotion of a healthy lifestyle. Optimism may serve as a psychological buffer that reinforces persistence in maintaining health behaviors, particularly when the outcomes are delayed, by sustaining motivation through positive future expectations. This positive outlook helps individuals to view health behaviors as investments with cumulative benefits, encouraging consistent effort even when immediate rewards are lacking. As a result, optimism can reduce the likelihood of reverting to unhealthy habits, promoting a sustained commitment to long-term health goals and increasing resilience against setbacks in the health improvement journey (Carver et al., 2010).

## 6. Implications

The results of this study highlight the need to develop dispositions that enable the realization of a health-promoting lifestyle. Educational programs should incorporate training to enhance optimism and self-efficacy, tailored to address gender-specific needs. For instance, stress management workshops and goal-setting strategies could be integrated into health sciences curricular. It is particularly important in the education process of future health promoters, as they often model actions in their surroundings. Additionally, policymakers and public health experts can take these findings as a basis of introducing programs and implementing plans that focus on enhancing optimism and self-efficacy in young adults so that they opt for health-promoting and preventive behaviors in the future (Mohsin et al., 2023).

## 7. Limitations and Recommendations for Future Research

In this study, due to the lack of Polish scales to diagnose specific self-efficacy in the context of health behaviors, only generalized self-efficacy was verified, which is a limitation of the work. Future research should be aimed at preparing Polish adaptations of scales that pertain to situation-specific beliefs connected with certain behaviors, e.g., exercise, diet, mental health prophylaxis, and sleeping patterns (Bill et al., 2023; Schwarzer, 1992).

Our study was also limited to verifying only the overall health behavior intensity. It would also be necessary to diagnose specific health behaviors (physical activity, nutrition, sleep quality, participation in preventive screenings, and stress management) using specific tools and then again verify their relationship with the intensity of dispositional optimism and self-efficacy. Such targeted research could provide more detailed insights into how these psychological dispositions influence various health behaviors, allowing for the development of more effective, personalized health promotion interventions. Additionally, reliance on self-reported data introduces potential biases, including self-presentation bias.

Additionally, this study involved a specific group of young adults—health sciences students. In this group, we may also expect a positive attitude toward a health-promoting lifestyle and greater knowledge about the health-promoting effects of various health behaviors, which is important in the process of implementing a health-promoting lifestyle, so the conclusions from this study can be generalized to adults with similar characteristics. Moreover, cultural factors specific to the Polish context may influence results, and caution should be exercised when generalizing findings to populations in different cultural or geographic settings.

Another limitation was the cross-sectional design, which limited the ability to establish causal relationships between self-efficacy, optimism, and health behaviors.

## 8. Conclusions

The study revealed that the vast majority of the examined students had at least a moderate level of general health behaviors (75%). Contrary to expectations, there was no significant gender difference in the intensity of general health behaviors. As expected, the frequency of health-promoting behaviors in various categories differed and varied by gender. The highest level of healthcare was observed in positive mental attitude, and the least in the proper eating habits subscale. Higher scores of PMA and PhA were more frequent among men, whilst women were characterized with higher PA.

Significant differences in the intensity of HB between students with low, average, and high levels of dispositional optimism appeared. Students with a high level of this disposition were more likely to exhibit high levels of healthcare compared to their less optimistic peers. When focusing on the category of behavior, LOT-R results turned out to be a statistically significant condition for higher frequency of positive mental attitude behaviors and preventive actions. The GSES level turned out not to differentiate health behavior intensities at a statistically significant level, but a tendency to take greater care of health in groups with a higher GSES result compared to students with a low level was observed.

Additionally, the results of this study paid attention to gender as a moderator variable of the relationships between HB and the examined dispositions. Men with a higher GSES level tended to be characterized by higher HB, and the opposite was true for women. Pro-health actions in men were the highest in those with a high level of optimism, whilst in women the opposite trend was observed. For the remaining areas of health behaviors, tendencies toward a different course of these relationships were observed.

In conclusion, this study underscores the critical role of optimism and self-efficacy in shaping health behaviors among health sciences students. Gender differences highlight the need for tailored interventions. Future longitudinal studies are recommended to validate these findings and explore the dynamic interplay of psychological dispositions in health behavior formation.

## Figures and Tables

**Table 1:** Health behaviors in the context of self-efficacy and gender.

Health Behaviors	Self-Efficacy	Gender	*M* ± *SD*	Effect	
General Health Behaviors (pts.)	Low level	Women	77.8 ± 11.4	Self-efficacy	*F*(2, 312) = 1.47, *p* = 0.231, η^2^p = 0.01
	Average level		79.9 ± 12.1	Gender	*F*(1, 312) = 1.22, *p* = 0.270, η^2^p < 0.01
	High level		77.8 ± 10.5	Self-efficacy*Gender	*F*(2, 312) = 2.41, *p* = 0.091, η^2^p = 0.02
	Low level	Men	77.3 ± 9.2		
	Average level		79.4 ± 10.3		
	High level		83.1 ± 8.8		
Positive Mental Attitude (pts.)	Low level	Women	19.1 ± 4.3	Self-efficacy	*F*(2, 312) = 2.76, *p* = 0.065, η^2^p = 0.02
	Average level		20.6 ± 3.8	Gender	*F*(1, 312) = 11.15, *p* < 0.001, η^2^p = 0.03
	High level		20.0± 3.2	Self-efficacy*Gender	*F*(2, 312) = 0.47, *p* = 0.626, η^2^p < 0.01
	Low level	Men	20.6 ± 2.8		
	Average level		21.6 ± 3.8		
	High level		21.8 ± 3.1		
Proper Eating Habits (pts.)	Low level	Women	18.9 ± 3.9	Self-efficacy	*F*(2, 312) = 0.57, *p* = 0.566, η^2^p < 0.01
	Average level		19.1 ± 4.7	Gender	*F*(1, 312) = 0.05, *p* = 0.827, η^2^p < 0.01
	High level		19.1 ± 4.3	Self-efficacy*Gender	*F*(2, 312) = 0.50, *p* = 0.608, η^2^p < 0.01
	Low level	Men	19.0 ± 2.9		
	Average level		18.7 ± 4.2		
	High level		19.8 ± 3.4		
Preventive Actions (pts.)	Low level	Women	20.7 ± 4.0	Self-efficacy	*F*(2, 312) = 1.37, *p* = 0.256, η^2^p = 0.01
	Average level		20.4 ± 4.5	Gender	*F*(1, 312) = 5.06, *p* = 0.025, η^2^p = 0.02
	High level		19.9 ± 3.9	Self-efficacy*Gender	*F*(2, 312) = 4.32, *p* = 0.014, η^2^p = 0.03
	Low level	Men	17.9 ± 3.1		
	Average level		19.1 ± 4.4		
	High level		20.8 ± 3.9		
Pro-health Activities (pts.)	Low level	Women	19.1 ± 4.2	Self-efficacy	*F*(2, 312) = 0.32, *p* = 0.727, η^2^p < 0.01
	Average level		19.7 ± 3.6	Gender	*F*(1, 312) = 4.52, *p* = 0.034, η^2^p = 0.01
	High level		18.8 ± 3.8	Self-efficacy*Gender	*F*(2, 312) = 1.21, *p* = 0.284, η^2^p = 0.01
	Low level	Men	19.8 ± 3.9		
	Average level		20.1 ± 4.0		
	High level		20.7 ± 3.5		

Notes: *M*, mean; *SD*, standard deviation.

**Table 2:** Health behaviors in the context of dispositional optimism and gender.

Health Behaviors	Dispositional Optimism	Gender	*M* ± *SD*	Effect	
General Health Behaviors (pts.)	Low level	Women	77.1 ± 11.2	Dispositional optimism	*F*(2, 312) = 4.22, *p* = 0.016, η^2^p = 0.03
	Average level		77.5 ± 11.8	Gender	*F*(1, 312) = 1.16, *p* = 0.283, η^2^p < 0.01
	High level		82.4 ± 10.5	Dispositional optimism*Gender	*F*(2, 312) = 0.52, *p* = 0.593, η^2^p < 0.01
	Low level	Men	78.9 ± 9.8		
	Average level		80.0 ± 9.9		
	High level		82.0 ± 9.4		
Positive Mental Attitude (pts.)	Low level	Women	19.0 ± 3.6	Dispositional optimism	*F*(2, 312) = 10.54, *p* < 0.001, η^2^p = 0.06
	Average level		19.8 ± 4.0	Gender	*F*(1, 312) = 10.87, *p* = 0.001, η^2^p = 0.03
	High level		21.5 ± 3.4	Dispositional optimism*Gender	*F*(2, 312) = 0.05, *p* = 0.951, η^2^p < 0.01
	Low level	Men	20.5 ± 4.0		
	Average level		21.2 ± 3.0		
	High level		22.7 ± 2.9		
Proper Eating Habits (pts.)	Low level	Women	19.5 ± 4.0	Dispositional optimism	*F*(2, 312) = 0.57, *p* = 0.564, η^2^p < 0.01
	Average level		18.7 ± 4.5	Gender	*F*(1, 312) = 0.03, *p* = 0.859, η^2^p < 0.01
	High level		19.1 ± 4.5	Dispositional optimism*Gender	*F*(2, 312) = 0.05, *p* = 0.949, η^2^p < 0.01
	Low level	Men	19.4 ± 3.3		
	Average level		18.9 ± 4.4		
	High level		19.2 ± 3.1		
Preventive Actions (pts.)	Low level	Women	20.1 ± 4.3	Dispositional optimism	*F*(2, 312) = 4.37, *p* = 0.013, η^2^p = 0.03
	Average level		19.9 ± 4.1	Gender	*F*(1, 312) = 4.21, *p* = 0.041, η^2^p = 0.01
	High level		21.4 ± 4.1	Dispositional optimism*Gender	*F*(2, 312) = 0.06, *p* = 0.944, η^2^p < 0.01
	Low level	Men	18.9 ± 3.9		
	Average level		19.0 ± 4.2		
	High level		20.5 ± 4.1		
Pro-health Activities (pts.)	Low level	Women	18.5 ± 4.1	Dispositional optimism	*F*(2, 312) = 1.15, *p* = 0.318, η^2^p = 0.01
	Average level		19.1 ± 3.9	Gender	*F*(1, 312) = 3.97, *p* = 0.047, η^2^p = 0.01
	High level		20.4 ± 3.2	Dispositional optimism*Gender	*F*(2, 312) = 3.57, *p* = 0.029, η^2^p = 0.02
	Low level	Men	20.1 ± 3.8		
	Average level		20.9 ± 3.1		
	High level		19.6 ± 4.5		

Notes: *M*, mean; *SD*, standard deviation.

## Data Availability

The data that support the findings of this study are available from the corresponding author (M.D.-J.) upon reasonable request.
